# Parallel trait adaptation across opposing thermal environments in experimental *Drosophila melanogaster* populations

**DOI:** 10.1111/evo.12705

**Published:** 2015-07-14

**Authors:** Ray Tobler, Joachim Hermisson, Christian Schlötterer

**Affiliations:** ^1^ Institut für PopulationsgenetikVetmeduni ViennaVeterinärplatz 1ViennaA‐1210Austria; ^2^ Vienna Graduate School of Population GeneticsVetmeduni ViennaVeterinärplatz 1ViennaA‐1210Austria; ^3^ Department of MathematicsUniversity of ViennaNordbergstrasse 151090ViennaAustria; ^4^ Max F. Perutz LaboratoriesDr. Bohr‐Gasse 91030ViennaAustria

**Keywords:** Experimental natural selection, fitness, temperature variation, thermal shock

## Abstract

Thermal stress is a pervasive selective agent in natural populations that impacts organismal growth, survival, and reproduction. *Drosophila melanogaster* exhibits a variety of putatively adaptive phenotypic responses to thermal stress in natural and experimental settings; however, accompanying assessments of fitness are typically lacking. Here, we quantify changes in fitness and known thermal tolerance traits in replicated experimental *D. melanogaster* populations following more than 40 generations of evolution to either cyclic cold or hot temperatures. By evaluating fitness for both evolved populations alongside a reconstituted starting population, we show that the evolved populations were the best adapted within their respective thermal environments. More strikingly, the evolved populations exhibited increased fitness in both environments and improved resistance to both acute heat and cold stress. This unexpected parallel response appeared to be an adaptation to the rapid temperature changes that drove the cycling thermal regimes, as parallel fitness changes were not observed when tested in a constant thermal environment. Our results add to a small, but growing group of studies that demonstrate the importance of fluctuating temperature changes for thermal adaptation and highlight the need for additional work in this area.

Determining which traits are involved in the adaptation of populations to new environments is a fundamental question in evolutionary biology. In moving to new environments, organisms are inevitably confronted by novel stressors, with thermal stress being particularly prominent (Hoffmann and Parsons 1991; Hoffmann and Hercus 2000; Bijlsma and Loeschcke 2005; Sørensen and Loeschcke 2007). Temperature affects diverse aspects of organismal physiology and metabolism and therefore represents a fundamental selective agent across all domains of life (Angilletta 2009). The ecological and evolutionary importance of thermal variation is readily observable in natural populations of ectothermic species whose geographical distribution is to a large extent determined by ambient temperature conditions (Andrewartha and Birch 1954).

The ectotherm *Drosophila melanogaster* has been the focus of numerous thermal evolution studies in both natural and experimental settings and has played a leading role in identifying which phenotypes are involved in thermal adaptation. Experimental evolution has proven to be invaluable to this end, allowing for specific thermal stressors to be investigated while simultaneously controlling demography and allowing replication. These studies can be broadly divided into two categories: those which use truncating selection on a stress‐related phenotype, in which a specific trait is directly enriched in successive generations by the researcher, and those which use a novel thermal environment to impose the selection pressure. Although the former method guarantees a rapid change in the selected phenotype, the latter method, often called “experimental natural selection” (Garland and Rose 2009), allows for the discovery of any trait that is critical for adaptation in new environments.

As small‐bodied ectotherms, *D. melanogaster* and other insects are able to maintain optimal internal thermal conditions by altering their behavior, that is, through behavioral thermoregulation, or by adjusting their performance at different temperatures, that is, by altering the thermal sensitivity of certain traits (Angilletta et al. 2002). Although behavioral thermoregulation appears to be important in natural *D. melanogaster* populations (Feder et al. 2000), opportunities for this are expected be limited in most experimental settings that typically employ constant temperatures and small spaces that allow for little thermal heterogeneity. Rather, experimental populations are expected to adapt by altering traits that optimize their sensitivity to their new thermal environments. Indeed, experimental *D. melanogaster* populations subjected to different constant temperatures show a range of putatively adaptive responses, with populations from hotter temperatures tending to have increased resistance to acute heat stress, decreased body size, and delayed development (Huey et al. 1991; Cavicchi et al. 1995; James and Partridge 1995; James et al. 1997). The adaptive nature of these responses is reinforced by geographical clines for each of these traits in natural *D. melanogaster* populations, including counter gradients for acute heat and cold stress resistance, which show genetic causation when assessed under laboratory conditions (Coyne and Beecham 1987; James et al. 1995, 1997; Hoffmann et al. 2002).

The most direct means of measuring adaption, however, is to quantify the fitness of evolving populations. Two important components of fitness in experimental Drosophila populations are larval competiveness and viability (Lewontin 1955; Mather and Caligari 1981; De Miranda and Eggleston 1988). Such measurements can be obtained by directly competing different experimental populations; however, this is typically not possible in most studies due to the absence of population‐specific markers. Nonetheless, robust measures of larval competitive ability and viability have been obtained previously for experimental Drosophila populations by assessing the relative performance of wild‐type larvae competed against a mutant strain (e.g., Santos et al. 1992; Kraaijeveld and Godfray 1997; Mery and Kawecki 2003; Trotta et al. 2007; Vijendravarma et al. 2009). Moreover, previous work has demonstrated that good estimates of the average Darwinian fitness of whole populations can be obtained by combining larval competition with fecundity measures into a single assay (e.g., Knight and Robertson 1957; Hartl and Jungen 1979; Haymer and Hartl 1983; Yamazaki 1984; Nuzhdin et al. 1995). Notably, such methods have only rarely been used to quantify fitness of Drosophila populations evolving at different temperatures (e.g., James and Partridge 1998; Santos et al. 2006).

Here, we use such a multicomponent fitness assay to determine whether two experimental *D. melanogaster* populations were adapting to opposing thermal environments that cycled daily between benign and stressful temperatures. Because the two environments differed primarily by the stressful temperature—either heat or cold stress—we predicted that populations would become better adapted to their own environments but not to the other. By extrapolating from results from other thermal natural selection studies (i.e., Huey et al. 1991; Cavicchi et al. 1995; James and Partridge 1995; James et al. 1997), we further hypothesized that selection would lead to improved stress resistance for the matching extreme temperature, and worse performance for the opposite stress, and slower (faster) developing, smaller (larger) individuals in the hot (cold) environment. However, while both populations were adapting to their respective environments, these traits tended to change in parallel rather than diverging through time. Furthermore, our results implicated the rapid daily temperature changes as the common stressor. Thus, our study demonstrates that temperature fluctuations represent an important and underappreciated selective agent that may contravene expectations based on constant thermal environments.

## Methods

### EXPERIMENTAL POPULATIONS

Female *D. melanogaster* were collected from Póvoa de Varzim, Portugal in July 2008 (Orozco‐terWengel et al. 2012). Following the collection, females were maintained in the laboratory as 113 isofemale lines for five generations, at which point they were used to create 10 mass‐bred replicate “base” populations. Each replicate base population comprised five females from each isofemale line (i.e., 565 females per replicate). The brief time between the collection and the establishment of the base population was used to limit the effects of drift within isofemale lines, thereby preserving much of the original segregating variation and decreasing the extent of linkage disequilibrium (LD) in the base population caused by amplifying inbred isofemale lines (also see Discussion). Five of the replicated base populations were thereafter maintained in either a “hot” or “cold” environment (see Experimental Environments). Replicate populations were maintained at approximately 1000 individuals, with approximately 50:50 sex ratios. Generations did not overlap; adults from the previous generation were collected following the peak of eclosion, randomly distributed into five new bottles and left to oviposit for two days, then transferred into another set of five new bottles for two more days of oviposition prior to being frozen for DNA storage. To prevent implicit selection for early fecundity, new generations were restricted to offspring from the second transfer when possible. Each replicate population was maintained in five 300 mL plastic bottles with 70 mL standard medium for the first 37 (23) generations in the hot (cold) environment, after which they were maintained in 170 mL plastic bottles with 40 mL of standard medium. Preliminary trials revealed that bottle size had no impact on the eclosion probability for density‐controlled populations (based on egg transfers). Furthermore, generation times remained consistent throughout the experiment (approximately two and four weeks in the hot and cold environments, respectively). Thus, consistent general population dynamics were maintained in each environment throughout the experiment, and this was not affected by the change in bottle size.

### EXPERIMENTAL ENVIRONMENTS

Both the hot and cold environments maintained a bimodal temperature and light regime on a repeated 12:12 h cycle. Temperatures cycled between 18°C and 28°C, or 10°C and 20°C, in the hot and cold environment, respectively, with the light (dark) period coinciding with the hot (cold) phase. These temperatures were chosen because prolonged exposure to temperatures below 10°C or above 28°C are known to decrease fecundity and viability in European *D. melanogaster* (Pétavy et al. 2001; Chakir et al. 2002), whereas the two intermediate temperatures (i.e., 18°C and 20°C) are comparatively benign. The natural climate of the experimental founder populations is typified by mild temperatures and moderate diurnal temperature fluctuations throughout the year (Fig. S1).

By placing thermochronometers (Davidson et al. 2003) inside the bottles used in the experiment—with one encased in the food and another in the air near the top of the bottle—we were able to estimate the rate of cooling and heating that was experienced by the larvae and adults during the experiment. Heating was particularly fast, with 85% of temperature change taking approximately 30 min in the air, and approximately 60 min in the food, for both environments (Fig. S2). Cooling was slower, but still relatively rapid, with 85% of temperature change taking approximately 45 min in the air, and approximately 75 min in the food, for both environments. The last 15% (i.e., 1.5°C) of change typically took 1–2 h longer due to the smaller temperature difference inside and outside the bottle as the two environments approached thermal equilibrium.

### RECONSTITUTED BASE POPULATION

To quantify and polarize the evolutionary changes during the experiment, the two evolving populations were compared to the original founder (base) population. Because we wanted to avoid any possible confounding environmental influences on the phenotypes due to assaying across different time points (see Controlling for Confounding Trait Variation), this required reconstituting five biological replicates of the base population from the isofemale lines in an identical fashion to the original base population as described above. The reconstituted populations should be good proxies of the original base population, providing that the effects of any genetic changes that have accrued within each isofemale line are random with respect to the two experimental environments. This seems to be a reasonable assumption for our experiment, given that the lines were maintained at low to moderate densities (approximately 20–100 individuals/vial/generation) and a temperature (18°C) that should be nonstressful for cosmopolitan *D. melanogaster* (Chen et al. 2015). Under these conditions, selection should be relatively ineffective and drift should dominate within lines. Furthermore, only three isofemale lines (out of 113) became extinct during the study. This would have caused only minor deviations in allele frequencies and suggests that any lost variants were rare, whereby the average trait values should be largely unchanged. Finally, although the isofemale lines would have become increasingly inbred during the experiment—which has been associated with reduced thermal stress resistance in *D. melanogaster* (Ehiobu et al. 1989; Dahlgaard and Hoffmann 2000; Pedersen et al. 2005)—our experimental design meant that the reconstituted base population flies were essentially F1 crosses between different isofemale lines (see below). This should effectively mask the aggregate effects of inbreeding depression within lines. Further appraisal of the impact of this method on the interpretation of our findings is provided in the Discussion.

### PHENOTYPIC ASSAYS

A series of phenotypic assays was performed on the evolving populations from the hot and cold environments—henceforth referred to as the hot population and cold population, respectively—along with a reconstituted base population. The following general trait categories were assayed: (1) acute thermal stress resistance, (2) body size, (3) developmental time, and (4) fitness. The first three have a long history of investigation in *Drosophila* thermal adaptation research and can be measured in a large‐scale parallel fashion, whereas fitness has rarely been directly quantified. All assays were performed after two generations in a shared environment (i.e., common garden environment, CGE; see below). Serial transfers of first‐generation adults were used to generate a large number of second‐generation CGE adult flies for phenotyping. Unless stated otherwise, each assayed trait used a separate transfer of the second‐generation CGE flies. All adult flies were aged between two and seven days when assayed, thereby closely matching the age range of flies used to seed new generations of the experimental environments (and thereby being most relevant for adult fitness). For all traits other than fitness, parallel assays were performed at two different time points during the experiment—(1) F34 cold and F59 hot and (2) F43 cold and F75 hot—with each utilizing an independently reconstituted base population. Fitness was only assayed for the later set of generations.

### CONTROLLING FOR CONFOUNDING TRAIT VARIATION

To improve our estimation of any adaptive phenotypic response, we sought to minimize the contribution of potentially confounding variables by performing the following three steps:

1. Prior to the phenotypic assays, all populations were subjected to two generations in a CGE that consequently standardized both the environmental and trans‐generational effects. Populations in the CGE were maintained under the standard culture conditions used in the experimental environments with 12‐h light:12‐h dark cycles that were synchronized across all environments. Four different common garden experiments were conducted, two at each of the cycling temperatures (18–28°C and 10–20°C) and two more at the mean temperature of each environment (i.e., 15°C and 23°C). Fitness traits were assayed in both cycling environments and the 23°C environment, while all other traits were limited to the two constant temperature environments. The choice of a single temperature for nonfitness traits was motivated by the desire to minimize any potential phenotypic variation that may have arisen due to development occurring within the bimodal experimental temperature regime.
2. To control for any trait‐specific effects of differing larval densities, a fixed number of eggs (approximately 200) were used to seed the second generation of each CGE for all populations—that is, the hot, cold, and reconstituted base—and replicates. To do this, first‐generation CGE adults were transferred to 170 mL bottles with new food and allowed to oviposit overnight. On the following morning, sections of food containing the required number of eggs were cut out and transferred into new 170 mL bottles (also containing food). The remaining first generation CGE adults were later discarded, and the bottles containing eggs returned to the relevant CGE. Two hundred eggs per bottle can be considered a moderate density relative to that in the experimental environments, where we were typically able to recover >1000 adults from a single transfer (i.e., five bottles) for each replicate.
3. For a given CGE, the hot, cold, and reconstituted base populations were assayed in parallel. In other words, all replicates of the two evolved populations, along with five newly reconstituted base population replicates, were simultaneously subjected to all steps of the CGE and subsequent phenotypic assays. This last step ensures that all environmental variables were shared among the hot, cold, and base populations for a given CGE.

### THERMAL STRESS RESISTANCE

Two types of ecologically relevant thermal stress resistance were quantified: the time taken for adult flies to either become knocked down or to recover from an acute exposure to heat and cold stress, respectively (Berrigan and Hoffmann 1998; David et al. 1998). For knockdown time to heat stress, 12 adult flies were placed into a transparent 200 mL plastic tube (Greiner) that was then sealed with plastic wrap and placed into an incubator at 38.5°C. To alleviate possible desiccation, a damp 1 × 1 cm square of Whatman filter paper was added to each tube prior to the start of the assay. Knockdown time was measured as the time taken for individuals to leave their feet or become inactive. For recovery from cold stress, 12 adult flies were moved into an empty vial that was placed in an ice‐filled tray inside a 0°C freezer where they were kept for 4 h. Following this, they were transferred into an empty Petri dish in a room maintained at a constant 25°C (±1°C) where the time taken to return to a standing position was recorded. The number of individuals knocked down/recovered was recorded at the end of every minute, with each assay continuing until either 60 min had passed or all individuals were knocked down/recovered. For both stress phenotypes, eight sets of tubes/Petri dishes were assayed at a time. Flies from different populations, replicates, and sexes were assayed in separate tubes/Petri dishes, and were randomly distributed across assays. This was done in blocks for the heat stress assays to account for a weak (approximately 0.5° C), but persistent, thermal gradient across the incubator. Sexes were separated while anaesthetized with CO_2_, with the assays taking place two days later to ensure sufficient recovery from the adverse effects of this procedure (Nilson et al. 2006; Colinet and Renault 2012). Overall 48 individuals were assayed for each combination of CGE, population, sex, and replicate (i.e., approximately 3000 flies per assay).

### DEVELOPMENTAL TIME

As described above, the second‐generation CGE populations were established by leaving first‐generation CGE adults to oviposit in new food for 24 h after which approximately 200 eggs were collected, transferred to new food bottles, and returned to the relevant CGE. Developmental time was then measured as the time from the initial oviposition period to pupation time, that is, the larval development time (James and Partridge 1995). Pupae were counted everyday until no new pupae were observed. The pupae were not sexed. Four bottles were quantified in this way for each replicate in the first assay (F34 cold and F59 hot), and two in the second assay (F43 cold and F75 hot). Only the 23°C CGE was performed in the second assay.

### BODY SIZE

Wing and leg measurements are correlated with general body size in *D. melanogaster* (Karan et al. 1998; Partridge et al. 1999; Turner et al. 2011). For each CGE, replicate and population, tibia length and wing area were measured for 30 males and females (i.e., 1800 flies per assay). A single rear leg and wing were dissected for each assayed individual and attached to a standard slide via a strip of adhesive tape. These were then photographed using a digital camera (DFC 300 FX) attached to a Leica M205‐FA stereomicroscope and the resulting images analyzed using ImageJ software (ImageJ64, version 1.43; Schneider et al. 2012). Tibia length was measured as the distance between the adjoining tarsus and femur, and wing area was measured according to Gilchrist and Partridge (1999). All flies used for this assay were derived from the eclosed adults of the developmental time assays. Flies were stored in a −20°C freezer for several months prior to dissection.

### FITNESS

Previous work has shown that assays that combine key fitness components in experimental populations (i.e., female fecundity, larval competitiveness, and larval viability) are good proxies for the average Darwinian fitness of whole populations in Drosophila (Knight and Robertson 1957; Hartl and Jungen 1979; Yamazaki 1984). We followed a similar strategy to measure fitness for the hot (F75), cold (F43), and reconstituted base populations. For each of the hot, cold, and reconstituted base population, 40 gravid females were transferred to a 170 mL bottle along with 60 gravid white‐eye *D. melanogaster* “reference” females (w1118). These were left to oviposit for two days, after which they were removed and their progeny expected to compete for resources, whereby larval competiveness and viability, along with female fecundity, were captured by these assays. This was repeated twice for each replicate, population, and environment, such that there were 90 “subreplicates” in total. Following the emergence of the subsequent generation, fitness was determined in each subreplicate by quantifying the number of enclosing individuals that were wild‐type (i.e., progeny of base, hot, or cold population flies). To ensure that the vast majority of surviving progeny were scored, eclosed adults were collected every five days for 15 days after the first individual eclosed; that is, three counts were performed in total. Statistical analyses (see below) were performed by combining consecutive counts of eclosed individuals for days ≤10 and ≤15. Because the test results were qualitatively similar between the two categories, only the latter is reported. To expedite the counting, males and females were quantified together. Previous trial assays had revealed that there was no discernable fitness difference between the two sexes for any combination of population and environment (data not shown).

### STATISTICAL ANALYSES

Because we were primarily interested in determining whether the populations were evolving to their new environments, we tested two specific adaptive hypotheses, having first fitted a generalized linear mixed model (GLMM) to each trait (see below). First, we tested whether each pair of hot, cold, and base populations were different conditional on sex and CGE. Second, we investigated whether phenotype values differed across the two assayed generations for each of the evolved populations, again conditional on sex and CGE. Thus, the first contrast tested whether the three populations differ after accounting for sex and CGE effects. The second contrast tested whether the populations were continuing to evolve through time, which required performing tests on transformed trait values that accounted for confounding variation across assays (see GLMM Fitting). All contrasts used the fitted values from the full models; that is, model reduction was not performed to ascertain the best fitting set of independent variables. This was justified on the grounds that removing the nonsignificant factors from the GLMMs made no difference to the significance of the subsequent contrasts (given that the effects of the nonsignificant terms were small).

### GLMM FITTING

For all traits except fitness, the explanatory variables comprised two fixed factors, population (i.e., base, hot, or cold) and CGE (i.e., 15°C or 23°C), and replicate as a nested random factor. Sex was included as an additional fixed factor for both the thermal shock and morphological traits. A block factor was also included for the heat stress phenotype to account for a thermal gradient across the incubator. For the fitness traits, the CGE factor was replaced with a factor for the environment of the assay (i.e., 10–20°C, 18–28°C, or constant 23°C). As for the predictor variables, the thermal stress (knockdown time or recovery time) and the developmental time (day of pupation) data were all fitted with a gamma response distribution (i.e., inverse link), because this resulted in a better fit of the data than using either normal or lognormal distributions (results not shown). The morphological data (tibia length and wing area) were fitted with a standard Gaussian error function, and the fitness data with binomial response distribution (i.e., logit link), as the predicted variable was the proportion of the wild‐type adults. The fitted models contained all possible interactions between fixed factors, which therefore included three and four way interactions in some cases.

Initial screening of the raw data suggested that the measurements were affected by uncontrolled environmental variation for some traits (see Results). This meant that using the raw observed values to make comparisons between datasets that were collected at different time‐points—namely, different CGEs from the same population, or comparisons across the two assayed generations—could lead to spurious results. Hence, for each trait we transformed the predicted variable by dividing all observed values of the evolved populations by the mean base population value conditional on sex, CGE, and assayed generation (i.e., either the first or second set of assays). Because the base population is expected to be genetically homogeneous for both assays (see Reconstituted Base Population), between‐assay phenotypic variation for this population should be attributable to uncontrolled experimental variables. Consequently, dividing all data by the relevant base means should control for such variation and facilitate comparisons across the different assayed CGEs and generations. After transforming the data for each trait, GLMMs were refitted to the combined data for the two assayed generations, with an additional fixed explanatory variable included to capture any assay effects. Visual inspection of QQ plots and residual homoscedasticity indicated that the adjusted data fulfilled the assumptions of their respective response distributions (data not shown). Tables S1 and S2 provide the ANOVA results from the GLMMs fitted to the raw and adjusted data, respectively.

### SOFTWARE

All analyses were performed using R (version 3.0.1; R Core Team 2013). The GLMMs used the lme4 package (version 1.0‐4; Bates et al. 2013) with subsequent significance testing performed via the ANOVA function from the car package (version 2.0‐19; Fox and Weisberg 2011). Parameters were estimated using restricted maximum likelihood (i.e., the “REML” option). Contrasts used the phia package (version 0.1‐5; De Rosario‐Martinez 2013), with the “Holm” method (Holm 1979) being used to correct for multiple testing.

## Results

### FITNESS

**Figure 1:**
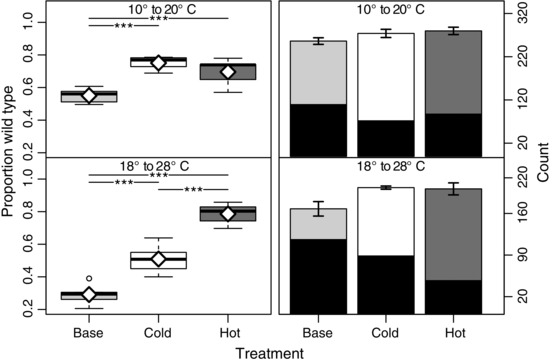
Boxplots showing the fitness of the hot (dark gray), cold (white), and base (light gray) populations quantified as the proportion of adult progeny (wild‐type) that eclosed after larval competition with an inbred white‐eye strain (left‐hand panels). White diamonds specify the mean values. The total number of eclosing wild‐type (coded as above) and white‐eye (black boxes) adults is shown in the adjacent barplots (right‐hand panels). Competition took place in each of the two experimental environments (cold, top panels; hot, bottom panels). Analyses were based on all adult flies observed to eclose for 15 days after the first observed eclosion. Significance for each pairwise comparison between populations is shown at the top of each panel (“.” ≤ 0.1, ‘‘*’’ ≤ 0.05, ‘‘**’’ ≤ 0.01, ‘‘***’’ ≤ 0.001).

As predicted, both evolved populations were significantly better adapted to their native environment than the base population. Unexpectedly, however, both evolved populations were also significantly fitter than the base population in their non‐native environment (Fig. 1, Table S3). For the hot population, the mean proportion of wild‐type flies increased by approximately 50 and 15% in the hot and cold environment, respectively, whereas the cold population increased by approximately 20% in both hot and cold CGEs. Thus, the evolved populations had the highest fitness in their native environments—although the difference between the evolved populations was only significant in the hot environment—implying that selection was driven by habitat‐specific and common factors.

Although many stressors common to both environments are conceivable, we suspected that the rapid temperature fluctuations in each environment were a major driver of the parallel adaptive response. The flies must tolerate the metabolic and physiological effects induced by these thermal fluctuations and are unlikely to have encountered such rapid changes in natural settings (see Discussion). To test whether the rapid temperature fluctuations were imposing a common stress in both environments, we exposed flies to a single fixed temperature, 23°C, while holding all other experimental parameters constant.

**Figure 2:**
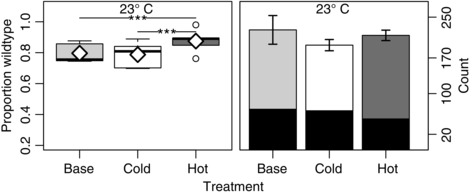
As for Figure 1 but with fitness assays carried out in a constant 23°C thermal environment. Analyses were based on all adult flies observed to eclose for 15 days after the first observed eclosion. Significance for each pairwise comparison between populations is shown at the top of each panel (“.” ≤ 0.1, “*” ≤ 0.05, “**” ≤ 0.01, “***” ≤ 0.001).

Indeed, the differences between the base and evolved populations were substantially reduced in this constant environment (Fig. 2, Table S3). The hot populations now exhibited a small, but significant, increase in fitness relative to the cold and base populations, whereas the difference between the cold and base populations was negligible.

Finally, because the number of eclosed adult flies was similar for the three populations, most notably for the two evolved populations (Figs. 1 and 2), the fitness differences between the three populations were not simply the result of differential female fecundity. Rather, fitness appeared to be largely driven by differences in larval competiveness and viability between the three populations.

### THERMAL STRESS RESISTANCE

**Figure 3:**
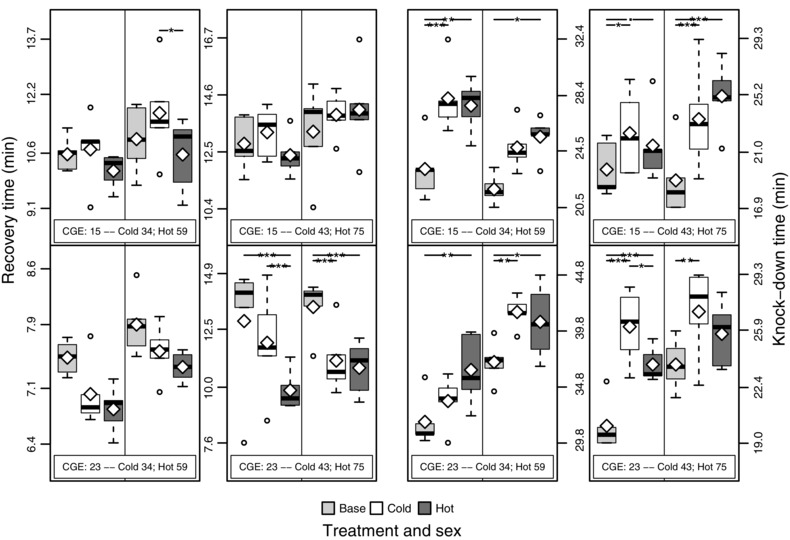
Box plot of recovery time (two left‐most columns) and knock down time (two right‐most columns) from acute cold and heat stress, respectively. Information on CGE and generation is inset in each panel, with females (males) on the left (right) of the central vertical line in each panel and population status shown in the key beneath the plot. The white diamonds specify the mean values. Significance for each pairwise comparison between populations is shown at the top of each panel (“.” ≤ 0.1, “*” ≤ 0.05, “**” ≤ 0.01, “***” ≤ 0.001).

Our expectation of habitat‐specific improvements in thermal stress resistance was not realized. Rather, both acute heat and cold stress resistance had a tendency to increase in both evolved populations relative to the base population, and this difference was significant for many of the contrasts (Fig. 3, Table S3). Cold stress resistance at 15°C CGE was a notable exception, with all three populations showing a similar level of resistance. This is indicative of a hardening effect at this temperature, which outweighed any selection on this trait. Notably, the two evolved populations tended to be similar to one another in all cases, regardless of sex or developmental temperature.

After correcting for the assay‐specific variation (see Methods), several sex–CGE combinations showed significant evidence for ongoing intergenerational change in both hot and cold populations (Fig. S3, Table S4). Of the significant combinations, the majority (6 of 9) had significantly increased stress resistance across generations, such that stress resistance was improving in most, but not all, cases.

### DEVELOPMENTAL TIME

**Figure 4:**
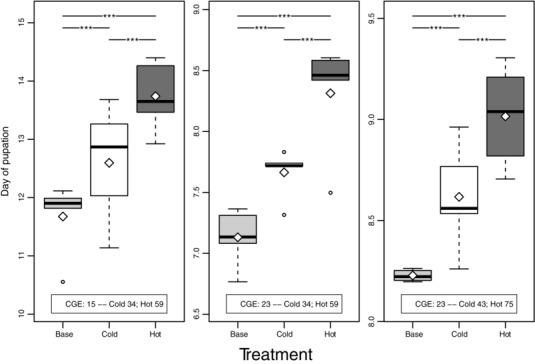
Box plot of developmental time. Information on CGE and the generations assayed are displayed within each panel. The *x*‐axis labels indicate the population. Note that there was no assay for the 15°C CGE in the second set of assayed generations. The white diamonds specify the mean values. Significance for each pairwise comparison between populations is shown at the top of each panel (“.” ≤ 0.1, “*” ≤ 0.05, “**” ≤ 0.01, “***” ≤ 0.001).

In keeping with results from a previous *D. melanogaster* experimental evolution study (James et al. 1997), the hot population evolved a significantly longer development time than the cold population in both CGEs and assayed generations. However, development time was significantly increased in both of the evolved populations when compared to the base, which contradicts our expectation of a divergent response for this trait (Fig. 4, Table S3). The scale of differences between treatments was sufficiently large—for example, nearly two days separated the base and cold populations in the 15°C CGE—to suggest that these patterns were unlikely to be a simple consequence of differential oviposition time or egg retention across the treatments during the 24 h egg laying period prior to assay. The difference in development time between the base and evolved populations began to decrease again between the two assayed generations (after correcting for assay‐specific variation) in the 23°C CGE, significantly so in the case of the hot population (Fig. S4, Table S4).

### BODY SIZE

**Figure 5:**
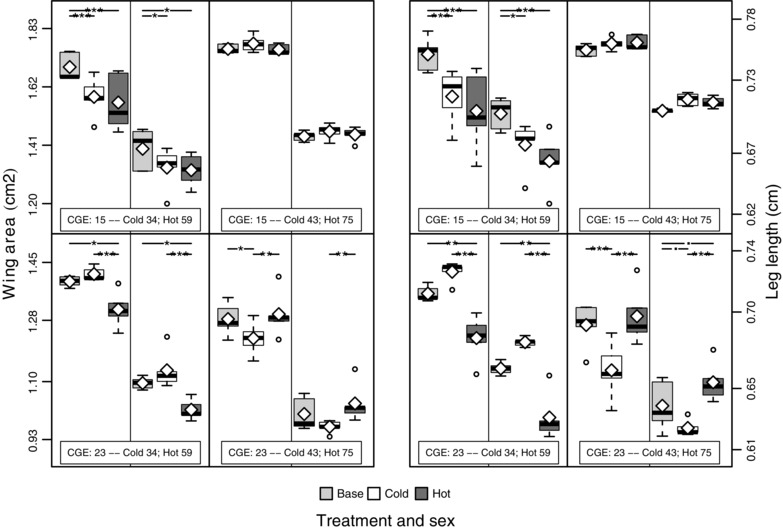
Box plot of wing area (two left‐most columns) and rear tibia length (right right‐most columns). Information on CGE and generation is inset in each panel, with females (males) on the left (right) of the central vertical line in each panel and population status shown in the key beneath the plot. The white diamonds specify the mean values. Significance for each pairwise comparison between populations is shown at the top of each panel (“.” ≤ 0.1, “*” ≤ 0.05, “**” ≤ 0.01, “***” ≤ 0.001).

The influence of temperature on plasticity in body size is well characterized—body size increases with decreasing developmental temperature (Kingsolver and Huey 2008)—and this pattern was replicated across all of our populations. However, the negative correlation between body size and temperature reported in previous Drosophila experimental evolution studies (Anderson 1972; Cavicchi et al. 1989; Partridge et al. 1994) did not evolve in our populations. Rather, no consistent trend emerged for either evolved population across the different time points (Fig. 5, Fig. S5, Table S3). Importantly, the variation in body size between the two different time points was considerably smaller for the base population than for the evolved populations. Furthermore, a strong positive correlation was observed between wing and leg dimensions (*r* = 0.688 and 0.687, based on all 1500 individuals for the first and second assays, respectively). This confirms the reliability of our measurements because these traits are known to be correlated (e.g., Robertson 1962; Turner et al. 2011) and suggests that these morphological traits were be reasonably robust to uncontrolled environmental variation introduced by our assaying procedure.

## Discussion

Our results clearly demonstrate that both of our experimental populations were adapting to their new environments, showing significant fitness gains relative to the base population. However, the expected responses were not observed for any trait; rather each phenotype either changed in parallel (e.g., thermal stress resistance and development), or fluctuated without any obvious pattern (e.g., morphology). Importantly, the consistency of the phenotypic response across replicates, in combination with the use of common environments for trait assays, strongly imply that selection—and not plasticity, trans‐generational effects, or drift—drove these changes. These findings imply that a common selective agent was a major driver of adaption in both evolved populations in conjunction with environment‐specific thermal stress. Possible explanations for these patterns are discussed in the following four sections.

### POTENTIAL BIAS INTRODUCED BY THE EXPERIMENTAL DESIGN

The observed parallel responses may have arisen if our phenotype measurements for the reconstituted base populations were systematically biased relative to those of the original base population. One possible source of bias comes from increasing inbreeding depression within the isofemale lines across the experiment. Nonetheless, our experimental design is expected to mask such effects by generating hybrids from inter‐isofemale line crosses (see Methods). Indeed, the similarity in fitness between base and evolved populations in the constant 23°C environment demonstrates that any putative within‐isofemale line inbreeding depression did not influence the fitness of the reconstituted base populations. Within‐isofemale line selection during the experiment represents another possible source of bias, as suggested by a previous study where thermal stress resistance responded to selection in *D. melanogaster* isofemale lines (Morrison and Milkman 1976). However, it is unclear why selection in the constant 18°C environment occupied by the isofemale lines would lead to decreased thermal resistance (or other trait changes) in both of the experimental environments. Furthermore, the adaptive responses in the Morrison and Milkman study were driven by strong truncating selection. We anticipate that similarly strong selection would be necessary for alleles to consistently escape stochastic loss from drift in our isofemale lines given their small population sizes. In the unlikely scenario that such a selected locus did consistently escape drift and had a measurable effect on the average phenotypes in our experimental conditions, laboratory selection stands out as a plausible candidate driver. Notably, such loci would ultimately lead to fitter reconstituted base populations in relation to the initial base population, making our estimates of fitness change more conservative overall. In summary, we consider the reconstituted base populations to be a robust basis for quantifying the trait changes during the experiment.

Another potential source of bias comes from the 15‐day interval of adult collection used in the fitness assays, which may have allowed for larval competition between slow developing larvae and the descendents of more rapidly developing individuals. Thus, because developmental rates had genetically diverged across the base, cold, and hot populations, larval competition may have been most intense for the faster developing populations, particularly in the hotter assay environments. Such differential larval competition may explain the parallel fitness increases observed in the hot (18–28°C) and cold (10‐20°C) environments, as well as the reduced number of adults in the former, but is incompatible with the results from the constant 23°C environment. Nonetheless, it is possible that development was generally slower in the 23°C environment than in the fluctuating hot environment, as developmental rates under constant temperatures can be retarded compared to cycling temperatures with the same mean in *D. melanogaster* (Siddiqui and Barlow 1972). Anecdotal observations indicate that such differences did not arise in our populations, however. Indeed, cycling thermal schemes similar to our own are known to result in comparable, or even delayed, development relative to constant environments ( Pétavy et al. 2001). In any case, the number of adults enclosing in both the constant 23°C and hot environments were very similar, which does not conform to the larger number expected had competition been relaxed in the constant environment. Consequently, our fitness measurements are unlikely to suffer from this type of competition‐induced bias and should reflect the true fitness patterns.

### LABORATORY ADAPTATION

When populations are maintained for multiple generations within a laboratory environment, they are inevitably exposed to novel conditions that may give rise selection on a variety of traits (Harshman and Hoffmann 2000; Matos et al. 2002; Huey and Rosenzweig 2008; Santos et al. 2010). This phenomenon, referred to as laboratory adaptation, has been thoroughly studied in *D. subobscura*, where it is known to drive convergent adaptive responses in life‐history traits, including increased early fecundity, larval competitiveness, and development time (Matos et al. 2000; Simões et al. 2008; Fragata et al. 2014). Results from *D. melanogaster* generally support these findings, with increased larval competitiveness, slower development, and larger body size all being reported for populations maintained in the laboratory over several generations (Frankham and Loebel 1992; Latter and Mulley 1995; Sgrò and Partridge 2000). Whether thermal tolerance traits are also subject to laboratory adaptation in Drosophila remains unclear, however. On the one hand, no change in either survivability or knockdown time under heat shock was found for *D. melanogaster* (Krebs et al. 2001) or sibling species *D. birchii* (Griffiths et al. 2005), respectively, following several generations of laboratory culturing. However, the latter population did evolve faster recovery to cold shock over the same culturing period (Griffiths et al. 2005). Although ecological drivers behind laboratory selection remain the subject of debate, increased larval densities and the restriction of oviposition to young adults—which are typical of *Drosophila* culturing methods, including our own—are likely suspects (Mueller 1991; Sgrò and Partridge 2000).

These results, in combination with the brief period that our flies spent in the laboratory prior to the start of the experiment, may imply that the parallel responses reported here were largely determined by laboratory adaptation. Nonetheless, different lines of evidence suggest that this was not true for all traits. First, the cold population often displayed significantly larger changes for body size and thermal stress traits than the hot population. Second, while developmental time initially increased for both evolved populations, it had started to decrease again when measured in the later generations. Thus, trait changes tended not to be proportionate to time spent in the laboratory, or were not strictly monotonic, contravening the expectations of laboratory selection. Finally, laboratory selection should have led to the evolved populations being fitter than the base population across all three environments in which fitness was assessed in this study, given that these environments shared all aspects of the culturing regime other than temperature. That this was not the case suggests that fitness was independent of the culturing conditions in our experiment (i.e., food conditions, transfer regime, adult age, larval densities, etc.). Hence, our results indicate that laboratory selection was not the sole driver of the parallel responses observed for all traits, with fitness being the most notable exception.

### ADAPTATION TO THERMAL FLUCTUATIONS

Two previous *D. melanogaster* experimental evolution studies showed that flies that had evolved in diurnally fluctuating thermal environments were fitter in both constant and fluctuating temperatures than those that had evolved under constant temperatures (Beardmore and Levine 1963; Long 1970). The authors attributed these fitness differences to adaptive plastic changes accruing in the populations that evolved to fluctuating temperatures, which improved their capacity to tolerate novel environments in general (i.e., not just those differentiated by temperature). Such plastic changes seem not to have evolved in our experimental populations, however, as the fitness differences between the evolved and base population were relatively minor in the constant environment. Rather, the environment‐specific fitness gains observed in our populations strongly imply that direct exposure to the thermal fluctuations was necessary to elicit the adaptive response. The combination of the swift rate and large amplitude of temperature change occurring twice daily in our experiment (i.e., 10°C change at approximately 0.15° to 0.25° C min^‐1^) is probably rarely experienced by natural *D. melanogaster* populations, including the progenitor population used here (see Fig. S1 for details). Thus, because our experimental populations comprise ectotherms that are unable to relocate away from the stressful temperatures, the rapid temperature fluctuations are a plausible candidate driver for the parallel fitness responses.

Whether the rapid thermal fluctuations were also responsible for the parallel changes observed in other traits remains unclear. One possibility is that the parallel increases in thermal stress resistance were a by‐product of selection for improved tolerance of the metabolic changes associated with both rapid heating and cooling. Thus, because the thermal stress assays will also induce brisk changes in internal body temperature in the captive flies, the evolved populations may have subsequently become more robust in these environments. Alternatively, selection for increased resistance to one type of stress can lead to correlated changes in other stress and life‐history related traits in *D. melanogaster* (e.g., Hoffmann and Parsons 1989; Bubliy and Loeschcke 2005). In particular, developmental time was observed to decrease in *D. melanogaster* populations selected for increased cold (Mori and Kimura 2008) and heat (Bubliy and Loeschcke 2005; Mori and Kimura 2008) resistance, whereas body size changes tend not to be correlated with thermal stress resistance (Anderson et al. 1991; Bubliy and Loeschcke 2005; also see Williams et al. 2012), echoing the results in our experiment. Selection for improved cold resistance is also known to produce more heat‐resistant flies, but not vice versa, in *D. melanogaster* (Bubliy and Loeschcke 2005; Mori and Kimura 2008). These results suggest that a correlated response to selection for improved thermal stress resistance may have been sufficient to drive some of the parallel phenotypic changes observed in our experimental populations.

The mechanistic basis underlying these putative intertrait correlations—for example, pleiotropy, LD between causal genes, or performance trade‐offs (Arnold 1992)—is not clear. A separate study revealed that moderate increases in LD had accrued in the hot population relative to the ancestral natural population after 67 generations of evolution, being almost uniform for single nucleotide polymorphisms (SNPs) separated by up to 50 Kb (Franssen et al. 2014). Because these LD differences represent the joint effects of selection and drift during the experiment, as well as the initial LD generated by amplifying inbred isofemale lines, the latter mechanism did not appear to have caused extensive LD in our base populations. However, selected haplotype blocks, some spanning several megabases, were inferred to rise from low to intermediate frequencies over 67 generations (Franssen et al. 2014). Consequently, the putative correlated phenotypic responses could have arisen in our study if multiple different functional alleles occupied the same rare selected haplotype blocks.

### ADAPTATION TO HABITAT‐SPECIFIC THERMAL STRESS

In addition to the parallel adaptive response, habitat‐specific fitness gains were also observed for both populations in their native environments, though only the hot population was significantly fitter. Reductions in viability (Chakir et al. 2002) and fertility ( Pétavy et al. 2001) were found to be much stronger at 10°C than 28°C in other European *D. melanogaster* populations, however, suggesting that habitat‐specific selection for thermal tolerance should have been stronger in the cold environment in our study. Although much of this apparent discrepancy could be due to the cold population having experienced many fewer generations of evolution, another potential explanation comes from a previous genomic analysis of these populations (Tobler et al. 2014). This study revealed that the majority of alleles that were selected over the first 15 generations of the experiment started at low frequencies in the hot environment, but were more likely to arise from intermediate to high frequencies in the cold environment. Based on the time of collection of the wild progenitor population, the authors concluded that this population had probably recently undergone selection for increased cold tolerance. Under this scenario, the base population would have preadapted the cold temperature, but relatively unfit under the hot temperature, used in the experiment. Thus, the observed habitat‐specific fitness patterns could have resulted from a combination of the reduced number of generations and partial preadaptation within the cold environment.

### COMPARISON WITH PREVIOUS GENOMIC STUDY

In contrast to the parallel phenotypic responses described in the current study, Tobler et al. (2014) found that the genomic response was largely environment‐specific over the first 15 generations of the experiment. In particular, the top candidates in the hot and cold populations were enriched for genes specifically associated with either heat‐ or cold‐stress resistance, respectively, but not *vice versa*, while genes conferring resistance to both stresses (which were mostly heat shock proteins; Hsps) were not enriched in either environment (Fig. S4). This discrepancy between the genomic and phenotypic results implies that the hot and cold populations may have followed different genetic pathways in adapting to the rapid temperature fluctuations. Alternatively, temperature‐specific selection may have dominated the early part of the experiment, whereas selection for tolerance to rapid temperature fluctuations may have been initially weaker, but eventually dominated fitness in the cold population in later generations. Either way, it seems unlikely that putative stress resistance genes, including Hsps, contributed to the shared fitness response observed for both evolving populations. Indeed, while Hsps facilitate general stress responses in *D. melanogaster* and other organisms (Feder and Hofmann 1999; Sørensen et al. 2003), long‐term or repeated expression of Hsp genes is known to be detrimental in *D. melanogaster* (Sørensen et al. 2003). Thus, it appears that alternative pathways may be favored to deal with rapid thermal oscillations in our experiment. Elucidation of these pathways will be a focus of future work.

### TEMPORAL THERMAL VARIATION AND ADAPTATION

A small but growing set of experimental studies have recently appeared that specifically address the role of temporal temperature variation on adaptation in *D. melanogaster*, either in environments where the temperature cycled across generations (Cooper et al. 2010, 2012; Yeaman et al. 2010) or within generations, but where the mean temperature increased linearly through time (Schou et al. 2014). In the latter study, resistance to desiccation and heat showed a negative correlation in fluctuating environments (Schou et al. 2014), opposing the positive association reported in studies conducted in constant environments (Hoffmann and Parsons 1989; Bubliy and Loeschcke 2005; Bubliy et al. 2012). Another recent study showed that *D. melanogaster* have an afternoon peak in locomotor activity when tested in cycling light and temperature environments, which was absent in environments where temperature (but not light) was uniform (Vanin et al. 2012). This revealed a previously unknown link between temperature variation and circadian rhythms in this species. Furthermore, simply imposing thermal variation during the development of *D. melanogaster* is also sufficient to produce a range of phenotypic changes (Economos and Lints 1986), including smaller body sizes (Pétavy et al. 2004; Kjærsgaard et al. 2012; Czarnoleski et al. 2013) and altered development (Pétavy et al. 2001), relative to populations that developed in constant thermal environments with the same mean temperature. Indeed, such plasticity may even be sensitive to the predictability of the thermal fluctuations (Manenti et al. 2014). Overall, these results demonstrate that expectations based on experimental populations that evolved in constant environments, and even for natural populations that are reared at constant temperatures, need not be readily generalizable to environments where temperature varies.

## Conclusions

The basic expectation of experimental natural selection—that populations should adapt to their new experimental environment—was fulfilled in our study. Our expectations of opposing trajectories of phenotypic change were not observed for any of the measured traits, however. Although it remains unclear to what extent laboratory selection drove these patterns, and whether the different traits were part of a correlated response, the evidence strongly implies that tolerance to rapid temperature fluctuations was a target of selection in each environment. This and a handful of other studies clearly demonstrate that temporal variation in thermal stress, at a variety of scales, may lead to contrasting phenotypic outcomes from studies where temperature is kept constant. Because of the default use of constant temperatures in most previous work, it remains largely unknown how important temporal variation is for shaping the adaptive response to thermal stress in *D. melanogaster* and other species. Considering that environmental heterogeneity is the norm in natural populations, it is clear that further work is needed to address these important issues.

## DATA ARCHIVING

The doi for our data is http://dx.doi.org/10.5061/dryad.r8mb0/1.

## Supporting information


**Supplemental Table S1**. Results of ANOVA (car R package, see methods) testing for all traits in both assayed generations (Cold 34 & Hot 59 or Cold 44 & Hot 75).
**Supplemental Table S2**. Results of ANOVA (car R package, see methods) testing for all traits except fitness after adjusting all values and adding the assayed generations as an additional fixed factor (Assay).
**Supplemental Table S3**. Results of contrasts (phia R package, see methods) testing all pairwise population differences given sex and CGE for all traits in both assayed generations (Cold 34 & Hot 59 or Cold 44 & Hot 75).
**Supplemental Table S4**. Results of contrasts (phia R package, see methods) testing for differences across the two assayed generations (Cold 34 & Hot 59 or Cold 44 & Hot 75) for each trait given population, sex and CGE. CGE = common garden experiment (constant 15% C, constant 23% C), Assay I = Cold 34 & Hot 59, Assay II = Cold 44 & Hot 75.
**Supplemental Figure 1**. Boxplot showing the maximum (red boxes), minimum (blue boxes), mean (green boxes) and range (gray boxes) of daily temperatures by month for the native habitat of the progenitor population Pòvoa de Varzim, Portugal.
**Supplemental Figure 2**.A 24hr time‐series showing the temperature cycles maintained in the hot (red) and cold (blue) experimental environments.
**Supplemental Figure 3**. Box plot showing the adjusted of recovery time (2 left‐most columns) and knock down time (2 right‐most columns) from acute cold and heat stress, respectively.
**Supplemental Figure 4**. Box plot of adjusted developmental time for hot (red) and cold (blue populations). The x‐labels denote the assay number (I = F34 cold & F59 hot, II = F43 cold & F75 hot).
**Supplemental Figure 5**. Box plot showing the adjusted of wing area (2 left‐most columns) and rear tibia length (4 right‐most columns) from acute cold and heat stress, respectively. Sex and population are listed in the panels, CGE and assay (I = F34 cold & F59 hot, II = F43cold & F75 hot) in the & labels.
**Supplemental Figure 6**. Enrichment of putatively selected SNPs from the cold (left panel) and hot (right panel) experimental populations in resistance genes unique to heat stress (red line), cold stress (blue line), or common to both stresses (black line).
