# Supplementary material for: Parallel trait adaptation across opposing thermal environments in experimental Drosophila melanogaster populations
Source: Evolution. 2015 Jul 14;69(7):1745–59. doi: 10.1111/evo.12705 (PMC4755034; doi:10.1111/evo.12705)
Supplement: Supplementary file 1 — Supplemental Table S1. Results of ANOVA (car R package, see methods) testing for all traits in both assayed generations (Cold 34 & Hot 59 or Cold 44 & Hot 75). Supplemental Table S2. Results of ANOVA (car R package, see methods) testing for all traits except fitness after adjusting all values and adding the assayed generations as an additional fixed factor (Assay). Supplemental Table S3. Results of contrasts (phia R package, see methods) testing all pairwise population differences given sex and CGE for all traits in both assayed generations (Cold 34 & Hot 59 or Cold 44 & Hot 75). Supplemental Table S4. Results of contrasts (phia R package, see methods) testing for differences across the two assayed generations (Cold 34 & Hot 59 or Cold 44 & Hot 75) for each trait given population, sex and CGE. CGE = common garden experiment (constant 15% C, constant 23% C), Assay I = Cold 34 & Hot 59, Assay II = Cold 44 & Hot 75. Supplemental Figure 1. Boxplot showing the maximum (red boxes), minimum (blue boxes), mean (green boxes) and range (gray boxes) of daily temperatures by month for the native habitat of the progenitor population Pòvoa de Varzim, Portugal. Supplemental Figure 2.A 24hr time‐series showing the temperature cycles maintained in the hot (red) and cold (blue) experimental environments. Supplemental Figure 3. Box plot showing the adjusted of recovery time (2 left‐most columns) and knock down time (2 right‐most columns) from acute cold and heat stress, respectively. Supplemental Figure 4. Box plot of adjusted developmental time for hot (red) and cold (blue populations). The x‐labels denote the assay number (I = F34 cold & F59 hot, II = F43 cold & F75 hot). Supplemental Figure 5. Box plot showing the adjusted of wing area (2 left‐most columns) and rear tibia length (4 right‐most columns) from acute cold and heat stress, respectively. Sex and population are listed in the panels, CGE and assay (I = F34 cold & F59 hot, II = F43cold & F75 hot) in the & labels. Supplementa [file EVO-69-1745-s001.pdf]

Table S1. Results of ANOVA (car R package, see methods) testing for all traits in both assayed generations (Cold 34 & Hot 59 or Cold 44 & Hot 75). Predictor variables are as follows. Pop = population (Base, Cold or Hot), Env = environment (18° to 28° C, 10° to 20° C or constant 23° C), CGE = common garden experiment (constant 15° C or constant 23° C). PosBlock = blocking term for position in shaker for heatshock assays (3 blocks = 2 rightmost, 4 central and 2 leftmost positions). The colon indicates interaction terms.

			Cold 34 : Hot 59		Cold 44 : Hot 75	
Trait	Predictor	Df	Chisq	P	Chisq	P
Fitness	Env	2	NA	NA	1876.8	<0.001
	Population	2	NA	NA	50.1	<0.001
	Env:Pop	4	NA	NA	717.4	<0.001
Heatshock	PosBlock	2	1.2	0.563	16.5	<0.001
	CGE	1	803.6	<0.001	82.6	<0.001
	Pop	2	23.3	<0.001	52.0	<0.001
	Sex	1	19.2	<0.001	39.8	<0.001
	CGE:Pop	2	0.9	0.648	9.1	0.011
	CGE:Sex	1	102.5	<0.001	0.1	0.708
	Pop:Sex	2	1.5	0.470	2.7	0.260
	CGE:Pop:Sex	2	7.7	0.022	14.1	<0.001
Coldshock	CGE	1	1155.7	<0.001	117.1	<0.001
	Pop	2	9.0	0.011	17.2	<0.001
	Sex	1	30.6	<0.001	16.5	<0.001
	CGE:Pop	2	12.5	0.002	98.4	<0.001
	CGE:Sex	1	2.6	0.109	2.2	0.139
	Pop:Sex	2	2.1	0.356	16.8	<0.001
	CGE:Pop:Sex	2	0.5	0.783	6.3	0.042
Development	CGE	1	25281.5	<0.001	NA	NA
	Pop	2	304.4	<0.001	88.7	<0.001
	CGE:Pop	2	52.6	<0.001	NA	NA
Wing area	CGE	1	1862.2	<0.001	9703.2	<0.001
	Pop	2	16.4	<0.001	2.5	0.286
	Sex	1	2256.4	<0.001	3686.8	<0.001
	CGE:Pop	2	76.2	<0.001	58.5	<0.001
	CGE:Sex	1	5.9	0.015	22.5	<0.001
	Pop:Sex	2	5.2	0.074	1.3	0.530
	CGE:Pop:Sex	2	2.5	0.281	0.8	0.684
Tibia length	CGE	1	140.1	<0.001	2265.8	<0.001
	Pop	2	24.4	<0.001	11.2	0.004
	Sex	1	745.6	<0.001	912.1	<0.001
	CGE:Pop	2	132.2	<0.001	94.3	<0.001
	CGE:Sex	1	3.0	0.084	1.4	0.232
	Pop:Sex	2	2.2	0.333	6.5	0.039
	CGE:Pop:Sex	2	1.6	0.439	2.4	0.299

Table S2. Results of ANOVA (car R package, see methods) testing for all traits except fitness after adjusting all values and adding the assayed generations as an additional fixed factor (Assay). Predictor variables are as follows. Pop = population (Base, Cold or Hot), CGE = common garden experiment (constant 15° C or constant 23° C), Assay = generation of evolved populations (i.e. Cold 34 & Hot 59, Cold 44 & Hot 75). PosBlock = blocking term for position in shaker for heatshock assays (3 blocks = 2 rightmost, 4 central and 2 leftmost positions). For developmental time (Development) measurements were limited to the 23° C CGE in the 2nd assayed generation and sex was not distinguished, such that a simpler model was fit to this data. The colon indicates interaction terms.

		Heatshock		Coldshock		Development		Wing area		Tibia length	
Predictor	Df	Chisq	P	Chisq	P	Chisq	P	Chisq	P	Chisq	P
PosBlock	2	2.5	0.281	NA	NA	NA	NA	NA	NA	NA	NA
Assay	1	8.2	0.004	18.7	<0.001	18.7	<0.001	295.6	<0.001	295.6	<0.001
CGE	1	9.5	0.002	212.4	<0.001	212.4	NA	31.6	<0.001	31.6	<0.001
Pop	1	0.1	0.820	12.6	<0.001	12.6	<0.001	1.1	0.289	1.1	0.289
Sex	1	0.0	0.870	3.7	0.056	3.7	NA	17.0	<0.001	17.0	<0.001
Assay:CGE	1	3.3	0.068	67.9	<0.001	67.9	NA	197.2	<0.001	197.2	<0.001
Assay:Pop	1	3.0	0.086	0.5	0.485	0.5	0.006	307.2	<0.001	307.2	<0.001
CGE:Pop	1	2.2	0.136	1.2	0.279	1.2	NA	0.3	0.567	0.3	0.567
Assay:Sex	1	1.9	0.167	0.7	0.415	0.7	NA	0.5	0.499	0.5	0.499
CGE:Sex	1	18.7	<0.001	5.2	0.022	5.2	NA	0.0	0.982	0.0	0.982
Pop:Sex	1	1.0	0.328	5.4	0.021	5.4	NA	1.1	0.288	1.1	0.288
Assay:CGE:Pop	1	5.3	0.022	15.4	<0.001	15.4	NA	242.6	<0.001	242.6	<0.001
Assay:CGE:Sex	1	42.6	<0.001	4.6	0.032	4.6	NA	8.4	0.004	8.4	0.004
Assay:Pop:Sex	1	3.9	0.047	14.2	<0.001	14.2	NA	2.5	0.116	2.5	0.116
CGE:Pop:Sex	1	4.0	0.046	3.5	0.062	3.5	NA	1.3	0.251	1.3	0.251
Assay:CGE:Pop:Sex	1	0.9	0.336	0.5	0.470	0.5	NA	2.9	0.086	2.9	0.086

Table S2. Results of ANOVA (car R package, see methods) testing for all traits except fitness after adjusting all values and adding the assayed generations as an additional fixed factor (Assay). Predictor variables are as follows. Pop = population (Base, Cold or Hot), CGE = common garden experiment (constant 15° C or constant 23° C), Assay = generation of evolved populations (i.e. Cold 34 & Hot 59, Cold 44 & Hot 75). PosBlock = blocking term for position in shaker for heatshock assays (3 blocks = 2 rightmost, 4 central and 2 leftmost positions). For developmental time (Development) measurements were limited to the 23° C CGE in the 2nd assayed generation and sex was not distinguished, such that a simpler model was fit to this data. The colon indicates interaction terms.

		Heatshock		Coldshock		Development		Wing area		Tibia length	
Predictor	Df	Chisq	P	Chisq	P	Chisq	P	Chisq	P	Chisq	P
PosBlock	2	2.5	0.281	NA	NA	NA	NA	NA	NA	NA	NA
Assay	1	8.2	0.004	18.7	<0.001	18.7	<0.001	295.6	<0.001	295.6	<0.001
CGE	1	9.5	0.002	212.4	<0.001	212.4	NA	31.6	<0.001	31.6	<0.001
Pop	1	0.1	0.820	12.6	<0.001	12.6	<0.001	1.1	0.289	1.1	0.289
Sex	1	0.0	0.870	3.7	0.056	3.7	NA	17.0	<0.001	17.0	<0.001
Assay:CGE	1	3.3	0.068	67.9	<0.001	67.9	NA	197.2	<0.001	197.2	<0.001
Assay:Pop	1	3.0	0.086	0.5	0.485	0.5	0.006	307.2	<0.001	307.2	<0.001
CGE:Pop	1	2.2	0.136	1.2	0.279	1.2	NA	0.3	0.567	0.3	0.567
Assay:Sex	1	1.9	0.167	0.7	0.415	0.7	NA	0.5	0.499	0.5	0.499
CGE:Sex	1	18.7	<0.001	5.2	0.022	5.2	NA	0.0	0.982	0.0	0.982
Pop:Sex	1	1.0	0.328	5.4	0.021	5.4	NA	1.1	0.288	1.1	0.288
Assay:CGE:Pop	1	5.3	0.022	15.4	<0.001	15.4	NA	242.6	<0.001	242.6	<0.001
Assay:CGE:Sex	1	42.6	<0.001	4.6	0.032	4.6	NA	8.4	0.004	8.4	0.004
Assay:Pop:Sex	1	3.9	0.047	14.2	<0.001	14.2	NA	2.5	0.116	2.5	0.116
CGE:Pop:Sex	1	4.0	0.046	3.5	0.062	3.5	NA	1.3	0.251	1.3	0.251
Assay:CGE:Pop:Sex	1	0.9	0.336	0.5	0.470	0.5	NA	2.9	0.086	2.9	0.086

Table S3. Results of contrasts (phia R package, see methods) testing all pairwise population differences given sex and CGE for all traits in both assayed generations (Cold 34 & Hot 59 or Cold 44 & Hot 75). Population = pairwise population comparison, CGE/Env = place of assay (either constant 15° C, constant 23° C, 18° to 28° C or 10° to 20° C).

					Cold 34 : Hot 59		Cold 44 : Hot 75	
Trait	Sex	CGE/Env	Population	Df	Chisq	P	Chisq	P
Fitness	Both	10 to 20	Base-Cold	1	NA	NA	54.5	<0.001
	Both	11 to 20	Base-Hot	1	NA	NA	28.9	<0.001
	Both	12 to 20	Cold-Hot	1	NA	NA	4.3	0.117
	Both	23	Base-Cold	1	NA	NA	4.1	0.117
	Both	23	Base-Hot	1	NA	NA	1.8	0.175
	Both	23	Cold-Hot	1	NA	NA	11.6	0.003
	Both	18 to 28	Base-Cold	1	NA	NA	27.1	<0.001
	Both	18 to 28	Base-Hot	1	NA	NA	151.5	<0.001
	Both	28 to 28	Cold-Hot	1	NA	NA	52.6	<0.001
Heatshock	Female	15	Base-Cold	1	15.6	<0.001	7.8	0.037
	Female	15	Base-Hot	1	12.7	0.004	5.8	0.081
	Female	15	Cold-Hot	1	0.1	1.000	0.1	0.709
	Male	15	Base-Cold	1	5.4	0.119	25.9	<0.001
	Male	15	Base-Hot	1	8.7	0.026	40.6	<0.001
	Male	15	Cold-Hot	1	0.4	1.000	1.8	0.394
	Female	23	Base-Cold	1	2.1	0.584	43.9	<0.001
	Female	23	Base-Hot	1	13.5	0.003	16.0	<0.001
	Female	23	Cold-Hot	1	4.7	0.149	7.0	0.049
	Male	23	Base-Cold	1	12.1	0.004	13.6	0.002
	Male	23	Base-Hot	1	8.2	0.029	4.8	0.112
	Male	23	Cold-Hot	1	0.4	1.000	2.3	0.394
Coldshock	Female	15	Base-Cold	1	0.1	1.000	0.8	1.000
	Female	15	Base-Hot	1	1.7	0.963	1.1	1.000
	Female	15	Cold-Hot	1	2.6	0.725	3.7	0.386
	Male	15	Base-Cold	1	3.2	0.573	2.1	0.755
	Male	15	Base-Hot	1	1.4	0.963	3.0	0.512
	Male	15	Cold-Hot	1	8.8	0.033	0.1	1.000
	Female	23	Base-Cold	1	4.7	0.264	4.8	0.222
	Female	23	Base-Hot	1	9.2	0.029	61.7	<0.001
	Female	23	Cold-Hot	1	0.8	1.000	32.3	<0.001
	Male	23	Base-Cold	1	2.2	0.832	29.8	<0.001
	Male	23	Base-Hot	1	5.2	0.224	41.3	<0.001
	Male	23	Cold-Hot	1	0.7	1.000	1.0	1.000
Development	Both	15	Base-Cold	1	29.7	<0.001	NA	NA
	Both	15	Base-Hot	1	131.0	<0.001	NA	NA
	Both	15	Cold-Hot	1	35.2	<0.001	NA	NA
	Both	23	Base-Cold	1	84.1	<0.001	22.3	<0.001
	Both	23	Base-Hot	1	326.7	<0.001	88.4	<0.001
	Both	23	Cold-Hot	1	78.8	<0.001	24.6	<0.001
Wing area	Female	15	Base-Cold	1	16.9	<0.001	0.9	1.000
	Female	15	Base-Hot	1	23.8	<0.001	0.0	1.000
	Female	15	Cold-Hot	1	0.6	1.000	1.1	1.000
	Male	15	Base-Cold	1	6.7	0.049	0.8	1.000
	Male	15	Base-Hot	1	8.6	0.023	0.1	1.000
	Male	15	Cold-Hot	1	0.1	1.000	0.3	1.000
	Female	23	Base-Cold	1	0.7	1.000	8.1	0.044
	Female	23	Base-Hot	1	9.9	0.013	0.5	1.000

	Female	23	Cold-Hot	1	15.9	<0.001	12.8	0.004
	Male	23	Base-Cold	1	2.2	0.553	3.6	0.534
	Male	23	Base-Hot	1	8.7	0.023	2.5	0.937
	Male	23	Cold-Hot	1	19.7	<0.001	11.9	0.006
Tibia length	Female	15	Base-Cold	1	16.1	<0.001	0.8	1.000
	Female	15	Base-Hot	1	29.1	<0.001	1.1	1.000
	Female	15	Cold-Hot	1	1.9	0.296	0.0	1.000
	Male	15	Base-Cold	1	8.8	0.015	2.6	0.725
	Male	15	Base-Hot	1	20.4	<0.001	1.4	1.000
	Male	15	Cold-Hot	1	2.4	0.296	0.2	1.000
	Female	23	Base-Cold	1	2.7	0.296	26.5	<0.001
	Female	23	Base-Hot	1	11.3	0.005	1.0	1.000
	Female	23	Cold-Hot	1	25.2	<0.001	37.8	<0.001
	Male	23	Base-Cold	1	4.0	0.183	6.4	0.094
	Male	23	Base-Hot	1	13.7	0.001	7.1	0.070
	Male	23	Cold-Hot	1	32.6	<0.001	26.9	<0.001

Table S4. Results of contrasts (phia R package, see methods) testing for differences across the two assayed generations (Cold 34 & Hot 59 or Cold 44 & Hot 75) for each trait given population, sex and CGE. CGE = common garden experiment (constant 15° C, constant 23° C), Assay I = Cold 34 & Hot 59, Assay II = Cold 44 & Hot 75.

Trait	Sex	Population	CGE	Assay	Df	Chisq	P
Heatshock	Female	Cold	15	I-II	1	5.5	0.078
	Female	Hot	15	I-II	1	7.5	0.038
	Male	Cold	15	I-II	1	6.5	0.055
	Male	Hot	15	I-II	1	13.6	0.002
	Female	Cold	23	I-II	1	35.2	<0.001
	Female	Hot	23	I-II	1	1.1	0.898
	Male	Cold	23	I-II	1	0.0	1.000
	Male	Hot	23	I-II	1	0.4	1.000
Coldshock	Female	Cold	15	I-II	1	0.7	1.000
	Female	Hot	15	I-II	1	0.1	1.000
	Male	Cold	15	I-II	1	0.6	1.000
	Male	Hot	15	I-II	1	15.3	<0.001
	Female	Cold	23	I-II	1	0.3	1.000
	Female	Hot	23	I-II	1	34.4	<0.001
	Male	Cold	23	I-II	1	26.9	<0.001
	Male	Hot	23	I-II	1	27.4	<0.001
Development	Both	Cold	23	I-II	1	2.1	0.146
	Both	Hot	23	I-II	1	39.8	<0.001
Wing area	Female	Cold	15	I-II	1	57.4	<0.001
	Female	Hot	15	I-II	1	57.9	<0.001
	Male	Cold	15	I-II	1	38.7	<0.001
	Male	Hot	15	I-II	1	37.5	<0.001
	Female	Cold	23	I-II	1	37.3	<0.001
	Female	Hot	23	I-II	1	51.4	<0.001
	Male	Cold	23	I-II	1	53.9	<0.001
	Male	Hot	23	I-II	1	109.6	<0.001
Tibia length	Female	Cold	15	I-II	1	80.1	<0.001
	Female	Hot	15	I-II	1	139.4	<0.001
	Male	Cold	15	I-II	1	70.3	<0.001
	Male	Hot	15	I-II	1	120.8	<0.001
	Female	Cold	23	I-II	1	112.6	<0.001
	Female	Hot	23	I-II	1	68.9	<0.001
	Male	Cold	23	I-II	1	67.6	<0.001
	Male	Hot	23	I-II	1	149.4	<0.001

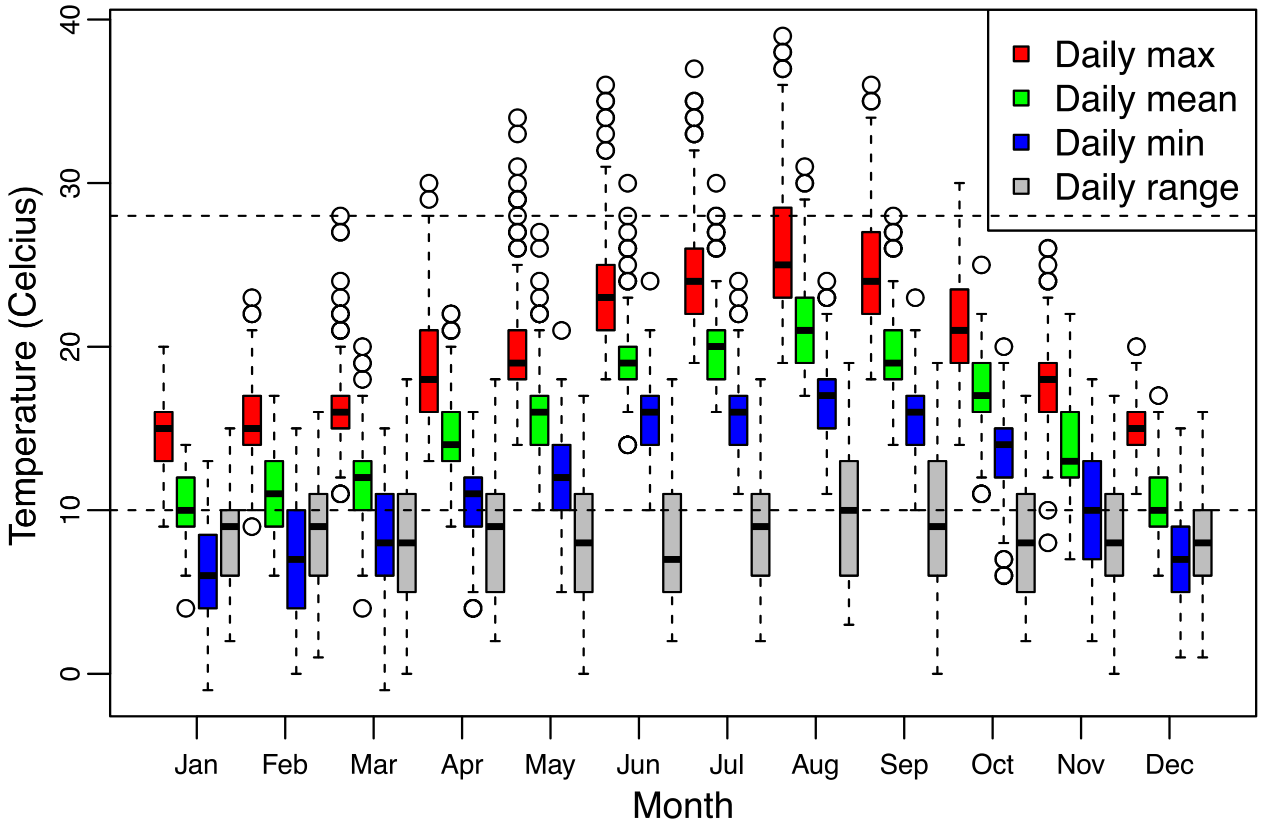

Figure S1. Boxplot showing the maximum (red boxes), minimum (blue boxes), mean (green boxes) and range (gray boxes) of daily temperatures by month for the native habitat of the progenitor population - Póvoa de Varzim, Portugal. The dashed black lines show the maximum and minimum temperatures in the experimental environments. The data is based on daily records collected between 01.08.2003 and 31.08.2008 – corresponding to the 5 years prior to the collection of the experimental founder lines – and was obtained from the Weather Underground website (http://www.wunderground.com/history/). Regarding the rate of temperature change in this area, we note that low temperatures typically occur shortly after sunrise, with high temperatures being reached few hours after midday (Ackerman and Knox 2013). Thus a conservative estimate of the maximum rate of temperature change in the Póvoa de Varzim area is on the order of ~0.067° $Cmin^{-1}$ (i.e. 16° C change over 4 hrs from 08:00 to 12:00, which is the highest observed temperature change from the month with the shortest period between sunrise and midday (December)). Despite being a conservative estimate, this is less than half of the lower limit of the rate of temperature change estimated in the experimental environments (see main text).

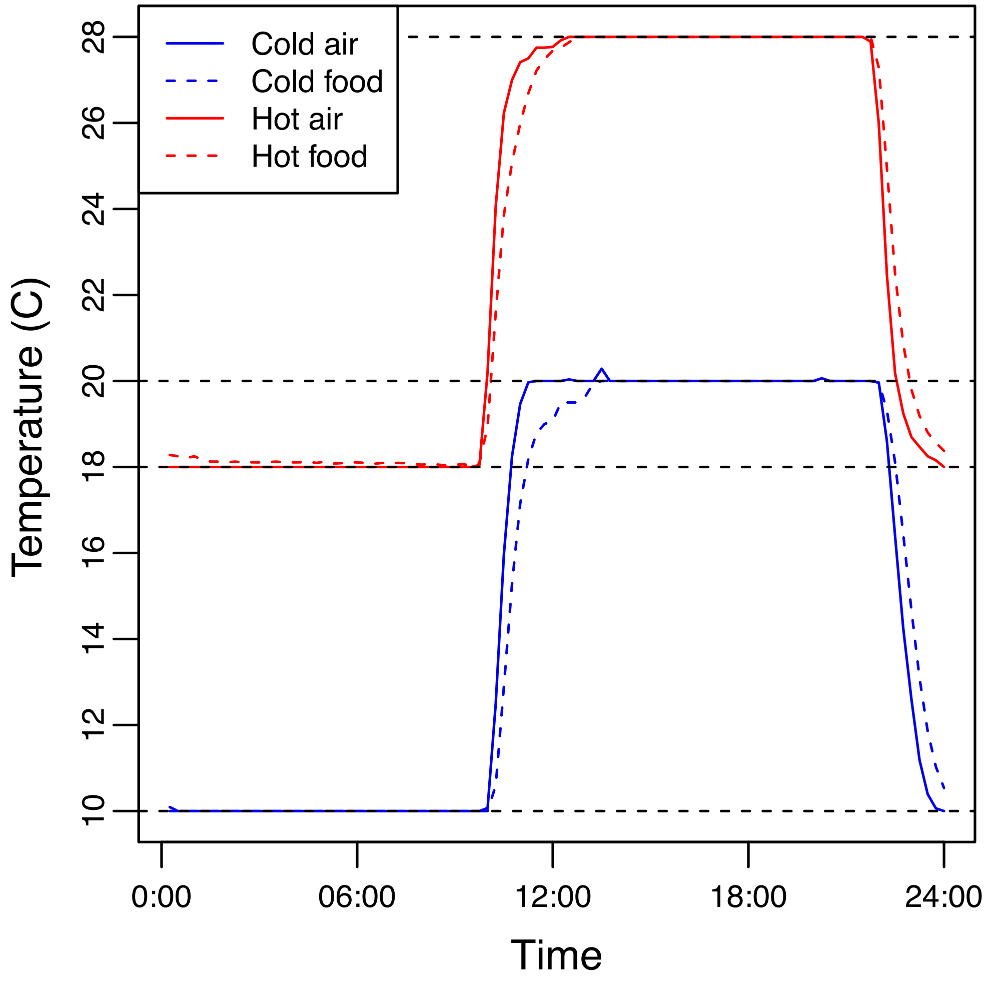

Figure S2. A 24hr time-series showing the temperature cycles maintained in the hot (red) and cold (blue) experimental environments. Data were collected using iButton® thermochronometers (see www.maximintegrated.com/en/products/comms/ibutton/DS1921G.html for model information). The measurements were made within bottles, with one iButton placed in the food at the base of the bottle (solid lines) and another adhered to the lid above the food (i.e. measuring air temperature in the bottle; dashed lines), thereby recording the temperatures experienced in the two mediums. Measurements were based on 2 bottles for each temperature regime (hot = 18° to 28° C, cold = 10° to 20° C). The dashed black lines show the modal temperatures for both environments. Temperatures were recorded every two minutes and the subsequent data were averaged over successive 15 minute intervals.

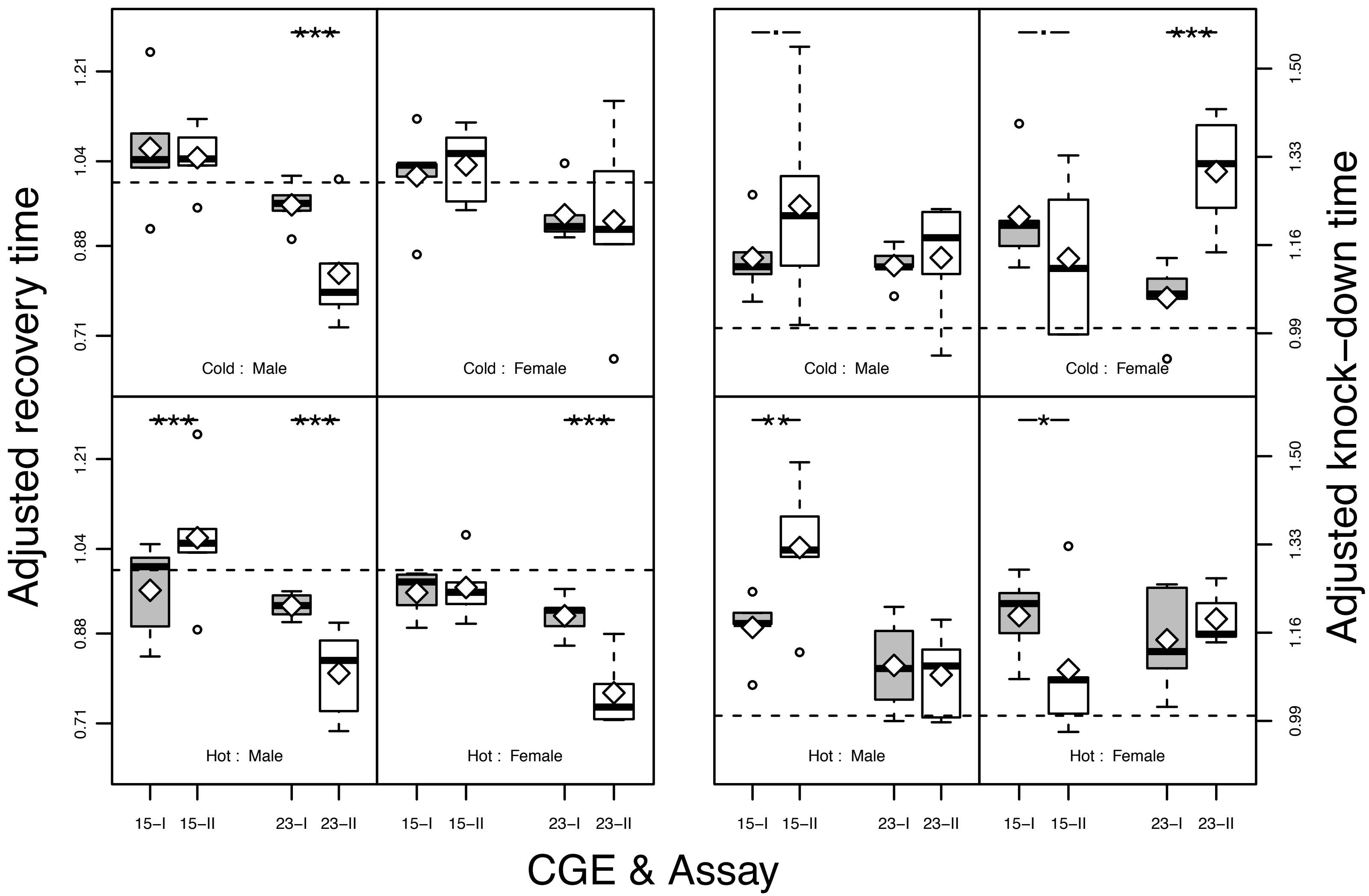

Figure S3. Box plot showing the adjusted of recovery time (2 left-most columns) and knock down time (2 right-most columns) from acute cold and heat stress, respectively. Sex and population are listed in the panels, CGE and assay (I=F34 cold & F59 hot, II=F43 cold & F75 hot) in the x-labels. Significance for the inter-assay comparisons is shown at the top of each panel. (“.” <= 0.1, “*” <= 0.05, “**”<= 0.01, “***” <=0.001)

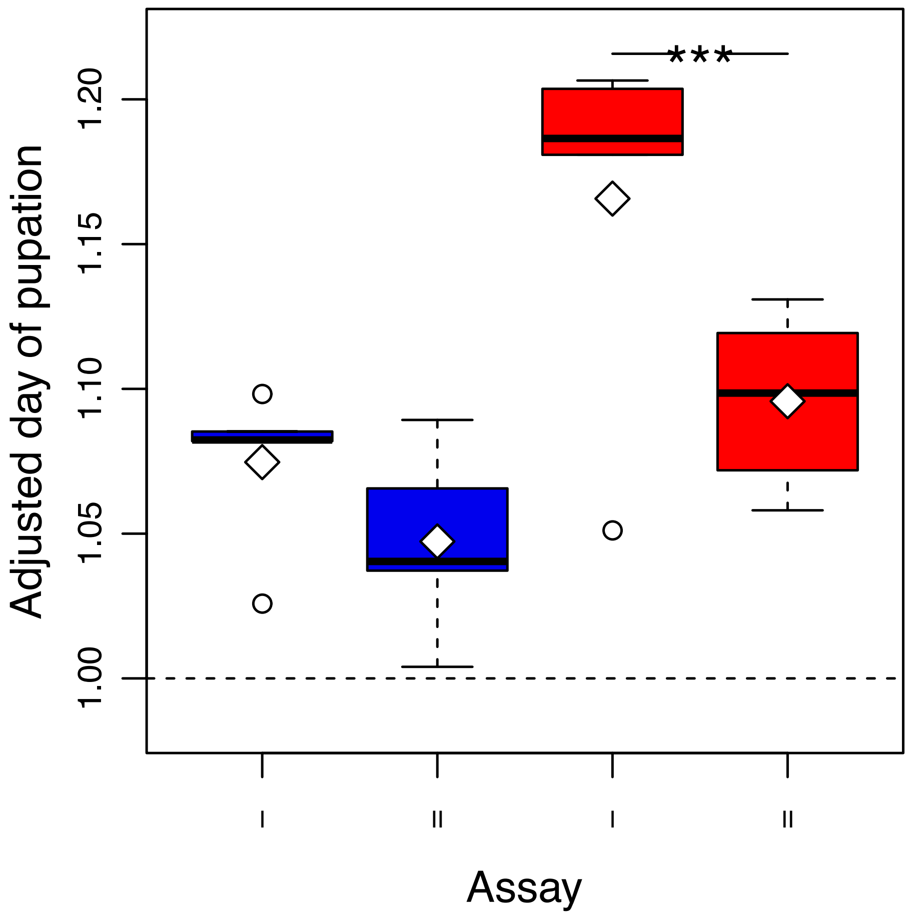

Figure S4. Box plot of adjusted developmental time for hot (red) and cold (blue populations). The x-labels denote the assay number (I=F34 cold & F59 hot, II=F43 cold & F75 hot). Only data for the 23° C CGE was available for both assayed generations. Significance for the inter-assay comparisons is shown at the top of each panel ("." <= 0.1, "*" <= 0.05, "**"<= 0.01, "***" <=0.001).

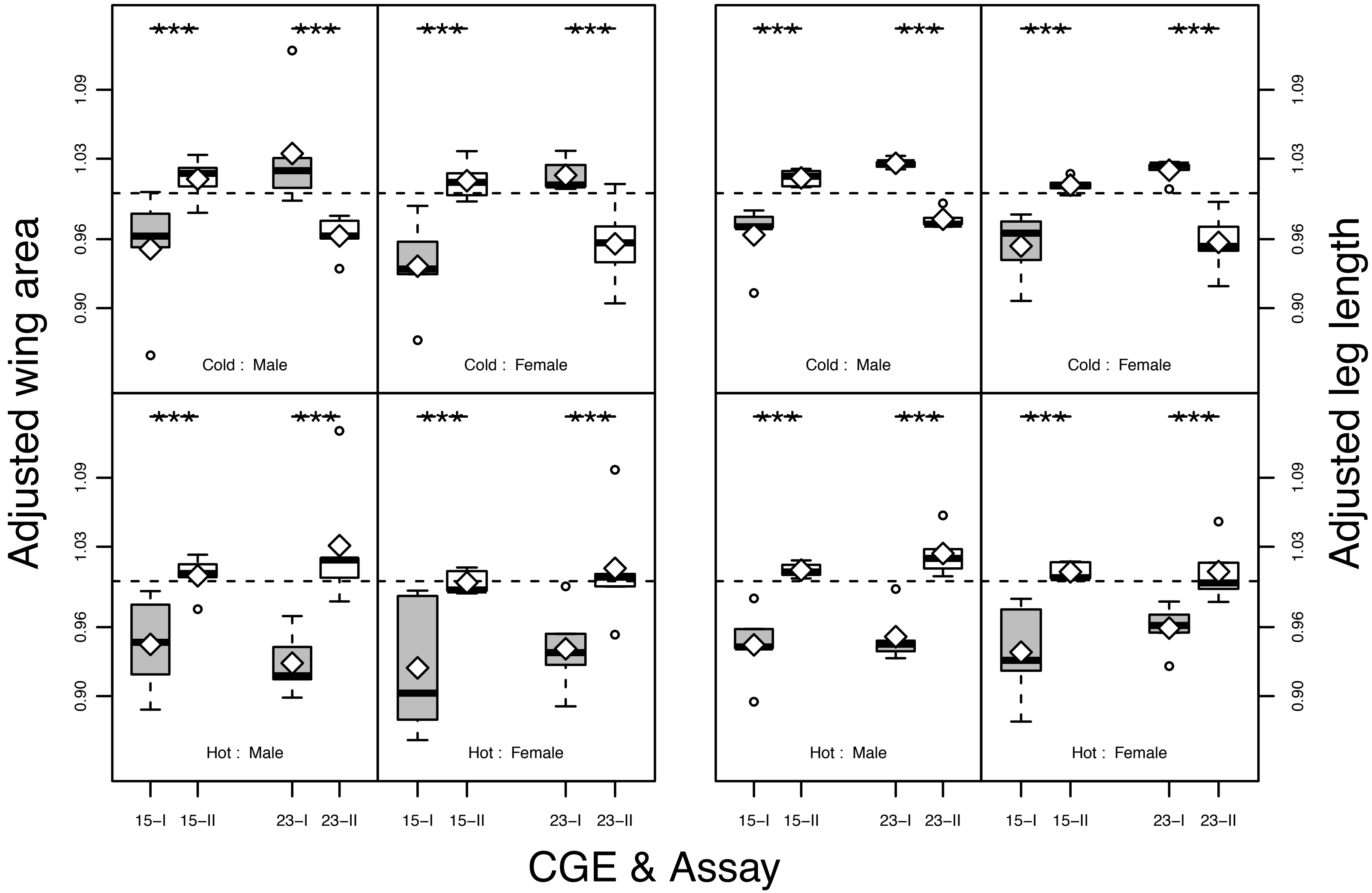

Figure S5. Box plot showing the adjusted of wing area (2 left-most columns) and rear tibia length (4 right-most columns) from acute cold and heat stress, respectively. Sex and population are listed in the panels, CGE and assay (I=F34 cold & F59 hot, II=F43 cold & F75 hot) in the x-labels. (“.” <= 0.1, “*” <= 0.05, “**”<= 0.01, “***” <=0.001).

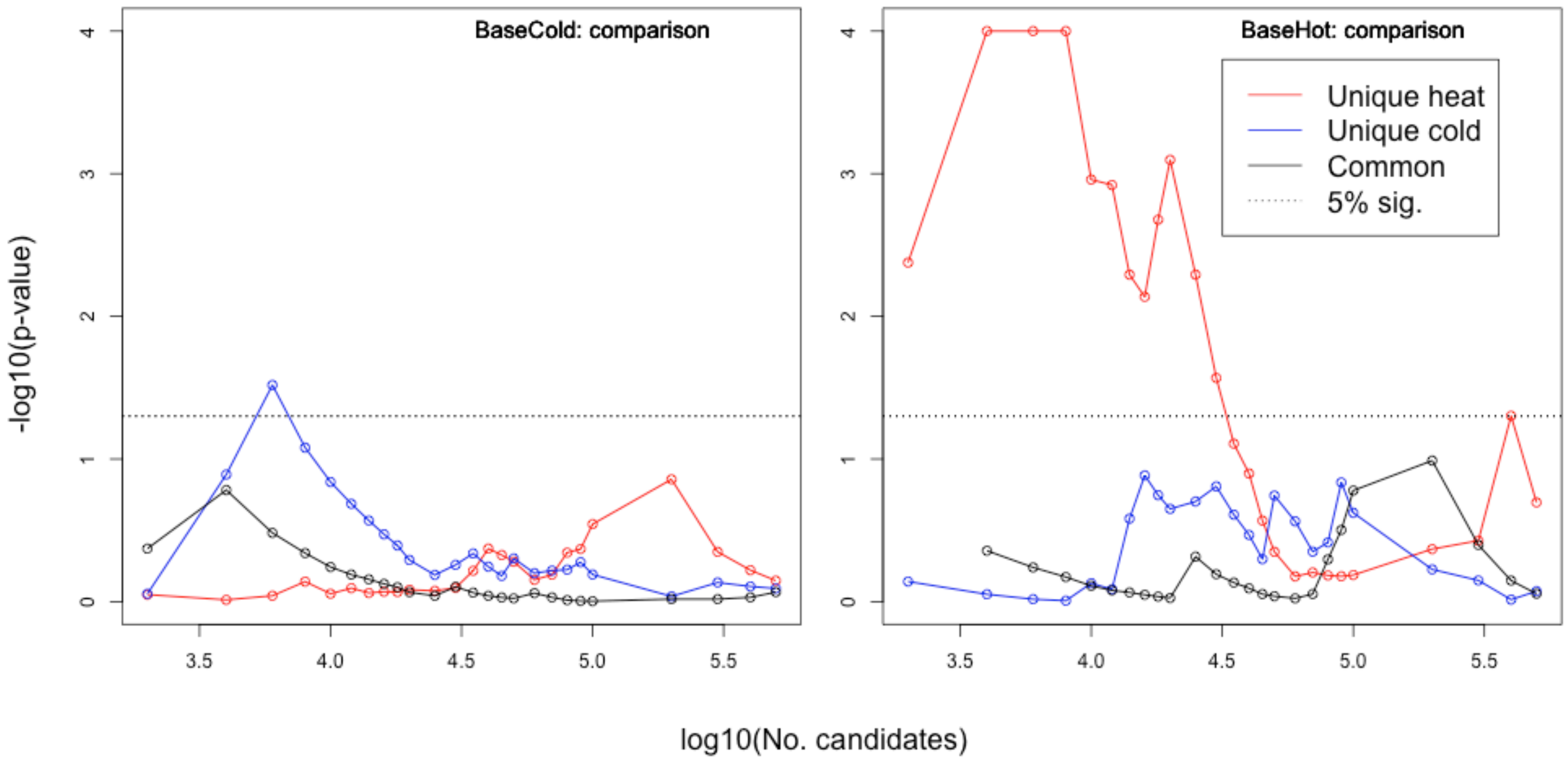

Figure S6. Enrichment of putatively selected SNPs from the cold (left panel) and hot (right panel) experimental populations in resistance genes unique to heat stress (red line), cold stress (blue line), or common to both stresses (black line). The common genes were taken as the intersection of heat and cold shock tolerance genes with significant associations from the CESAR database (http://cesar.org.au). The full common gene list comprised the following genes (CG names): "desat2" (CG5925), "Frost" (CG9434), "Glycerol 3 phosphate dehydrogenase" (CG9042), "Heat shock RNA omega" (CR31400), "Heat shock factor" (CG5748), "Heat shock protein 23" (CG4463), "Heat shock protein 26" (CG4183), "Heat shock protein cognate 4" (CG4264), "Heat shock protein 83" (CG1242), "mitochondrial acyl carrier protein 1" (CG9160), & "Pinocchio" (CG4710). See Tobler et al. (2014) for a detailed account of candidate SNP detection and enrichment tests, and for a full list of the unique genes.

Bibliography

Ackerman, S. A. And J. A. Knox. 2013. Meteorology. Jones & Bartlett Publishers.

Tobler, R., S. U. Franssen, R. Kofler, P. Orozco-terWengel, V. Nolte, J. Hermisson, and C. Schlötterer. 2014. Massive habitat-specific genomic response in *D. melanogaster* populations during experimental evolution in hot and cold environments. Mol. Biol. Evol. 31:364 – 375.
